# Intravenous administration of sodium propionate induces antidepressant or prodepressant effect in a dose dependent manner

**DOI:** 10.1038/s41598-020-77085-z

**Published:** 2020-11-16

**Authors:** Chunyan Hao, Zefeng Gao, XianJun Liu, Zhijiang Rong, Jingjing Jia, Kaiqi Kang, Weiwei Guo, Jianguo Li

**Affiliations:** 1 School of Chemical and Biological Engineering, Taiyuan University of Science & Technology, Taiyuan, 030021 China; 2 Key Laboratory of Chemical Biology and Molecular Engineering of Ministry of Education, Institutes of Biomedical Sciences, Shanxi University, No. 92, Wucheng Road, Xiaodian District, TaiyuanShanxi, 030006 China; 3 School of Life Science, Shanxi University, Taiyuan, 030006 China

**Keywords:** Experimental models of disease, Drug development, Neurological disorders

## Abstract

Propionate has been reported to exert antidepressant effects, but high-dose propionate may induce autism-like symptoms in experimental animals through induction of dysbiosis of neurotransmitters. The bi-directional effects of propionate seem to be dose-dependent. However, due to the pathological discrepancies between depression and autism, conclusions drawn from autism may not be simply transferable to depression. The effect and underlying action mechanisms of high-dose propionate on depression remains undetermined. To investigate the effects of propionate on depression, propionate dose gradients were intravenously administrated to rats exposed to chronic unpredictable mild stress (CUMS) for 1 week. Results of these behavioral tests demonstrate that low-dose propionate (2 mg/kg body weight/day) induces antidepressant effect through bodyweight recovery, elevated reward-seeking behaviors, and reduced depression-like behaviors, while high-dose propionate (200 mg/kg body weight/day) induces prodepressant effects opposite of those of low-dose propionate. A comprehensive profiling of neurotransmitters in the hippocampus demonstrated that CUMS induces reduction of NE (Norepinephrine), DA (Dopamine). GABA (γ-aminobutyric acid) was recovered by low-dose propionate, while high-dose propionate exerted more complicated effects on neurotransmitters, including reduction of NE, DA, 5-Hydroxytryptamine and Tryptophan, and increase of GABA, Kynurenine, Homovanillic acid, 3-hydroxyanthranilic acid, 3-hydroxykynurenine, 3,4-dihydroxyphenylacetic acid, and 3-methoxytyramine. The neurotransmitters disturbed by high-dose propionate suggest metabolic disorders in the hippocampus, which were confirmed by the clear group separation in PCA of metabolomic profiling. The results of this study demonstrate the double-edged dose-dependent effects of propionate on depression and suggest potential cumulative toxicity of propionate as a food additive to mood disorders.

## Introduction

Depression is a worldwide popular mood disorder, characterized by persistent low mood state and anhedonia^1,2^. Depression exerts great adverse effects on personal health and expends high costs of medical care^3^. Current first-line antidepressants, including tricyclic antidepressant (TCA)^4^ and selective serotonin reuptake inhibitors (SSRIs)^5^, are not efficacious against Major Depressive disorder (MDD)^6^. Only about 70% of people with depression exhibited improved mental states with standard pharmacological treatments^7^. Thus, there is an increasing need to find novel antidepressants^8^.

Short chain fatty acids (SCFAs), including acetate, propionate, and butyrate, have recently been shown to have antidepressant effects^9,10^. The antidepressant effects of butyrate have been confirmed repeatedly in multiple pathways, such as acting as an inhibitor of histone deacetylase (HDACi)^11^, restoring brain-blood-barrier impairments^12^, or altering the expression of the 1A receptor of 5-hydroxytryptamine (5-HT 1A ) in the hypothalamus^13^. Propionate was reported to exert antidepressant effects when administered individually^10^ or in combination with other short chain fatty acids (SCFAs)^14^.

Given its antidepressant effects, propionate administrated in high dose may induce neurotransmitter dysbiosis in the central nervous system, and has been applied for autism modelling in experimental animals^15,16^. The propionate-induced neurotransmitters dysbiosis^17,18^ involves depletion of key neurotransmitters (serotonin [5-HT], dopamine [DA], and gama-aminobyteric acid [GABA]), significant increased proinflammation (marked by tumor necrosis-α [TNF-α], and interlukin-6 [IL-6]) and pro-apoptosis (marked by caspase-3). However, the antidepressant effect of propionate also involves elevations of neurotransmitters^14^. The bidirectional effects of propionate on key neurotransmitters of the central nervous system led us to speculate that the action modes of propionate on depression may be dose-dependent and may be correlated with the metabolism of neurotransmitters in the central nervous system.

To validate the above speculations, we conducted short term intravenous administration of propionate dose gradients in rats exposed to chronic unpredictable mild stress (CUMS). We quantified the alterations of neurotransmitters, identified the propionate-associated metabolites, and proposed novel connections between high-dose propionate and neurotransmitters depletion in depression.

## Materials and methods

### Animals and reagents

Male Sprague–Dawley (SD) rats were purchased from Beijing Vital River Laboratories Co. (SCXK [Jing] 2016-006), weighting 200 g (± 10 g) on average. Four rats were housed per cage with free access to food and water, under controlled room humidity (45 ± 15%), temperature (25 °C ± 1 °C), and light (lights on at 7:30 a.m., 12-h day/night switch). The newly arrived rats were allowed to adapt to their new environments for one week.

Sodium propionate and neurotransmitter standards, including 5-hydroxytryptamine (5-HT), dopamine (DA), gama-aminobyteric acid (GABA), Norepinephrine (NE), Kynurenine (KYN), 5-hydroxyindole acetic acid (5-HIAA), 3,4-dihydroxyphenylacetic acid (DOPAC), 3-methoxytyramine (3-MT), 3-hydroxyanthranilic acid (3-HAA), 3-hydroxykynurenine (3-HK), were purchased from Sigma-Aldrich (St. Louis, MO, USA). Homovanillic acid (HVA) and derivatization reagent dansyl chloride were purchased from Tokyo Chemical Industry (Tokyo, Japan). Formic acid, methanol, acetone, and acetonitrile were obtained from Merck (Darmstadt, Germany). All solvents were HPLC grade or above.

### CUMS modelling

CUMS modelling was performed according to a previously described protocol^19^. Briefly, individually housed rats were subjected to one to four of the following stressors per day in a random order for 4 weeks: swimming in 4 °C water for 5 min, tail clamp for 2 min, foot-shock for 2 min (current density: 2 mA, shock for 4 s with an interval of 8 s), subject to room temperature at 45 °C for 5 min, water deprivation for 24 h, food deprivation for 24 h. Each rat was subjected to all of the above tests in a random order for unpredictable stressors exertion, and only received one test if the test lasted for 24 h. Samples of orbital blood were collected every week. The heparin sodium anticoagulant blood was centrifuged at 3000 rpm for 15 min, the supernatants were aliquoted and stored at -80 °C. The rats were anaesthetized with ethyl carbamate and sacrificed by femoral artery blood sampling after experiment completion. Brain dissection and hippocampus separation were performed on ice immediately after sacrifice of the rats. Hippocampal samples were weighted and stored at -80 °C for future use.

### Propionate administration

**Figure 1:**
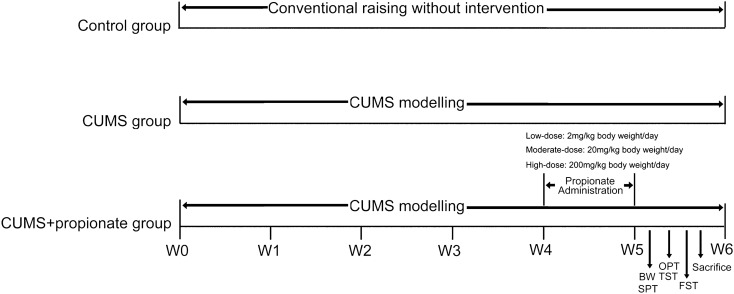
Experimental scheme of this study. Male Sprague–Dawley rats were divided into five groups: a control group, a CUMS group, a CUMS + low/moderate/high-dose propionate groups – each at 16 rats per group. The control group was conventionally raised and received no intervention. The CUMS group was exposed to chronic unpredictable mild stress (CUMS) during the entire process of the experiment. The CUMS + low/moderate/high-dose propionate groups were exposed to CUMS during the entire process of the experiment and received low/moderate/high-dose (2/20/200 mg/kg body weight/day) propionate during the fifth week of the CUMS modelling, respectively.

Rats were randomly divided into four groups (Fig. 1) of 16 rats per group. The CUMS group intravenously received 400 μl PBS per day. The CUMS + low/moderate/high-dose propionate groups intravenously received 2/20/200 mg sodium propionate in PBS solution per day, respectively. Intravenous propionate administrations were continuously carried out for one week after the success of CUMS modelling, with an interval of 24 h (Fig. 1).

### Body weight measurement

To reduce possible errors between each measurement, the body weight of each rat was measured at 8:30 a.m. on day 0, 7, 14, 21, 28, and 35 of the CUMS modelling.

### Sucrose preference test

A sucrose preference test (SPT) was used to assess hedonic-like behavior in the rats. Exposure to 1% sucrose solution for 24 h was performed before SPT to avoid neophobia. One bottle of 1% sucrose solution and another bottle of tap water were simultaneously provided to each rat for 4 h. Consumptions of water and sucrose were recorded, respectively. The sucrose preference rate (SPR) was calculated according to the following equation: SPR = sucrose consumption (g)/(sucrose consumption (g) + water consumption (g)).

### Tail suspension test

A tail suspension test (TST) was used to assess depressive-like behavior. Rats were hung by their tail using adhesive tape to a 30 cm-elevated grid bar for 6 min. A numeric tripod-fixed camera was applied for videotaping of the experiments. The time spent immobile was recorded. A pre-test was performed before any formal test to allow rats to adapt to the newly faced challenge.

### Open field test

An open field test (OPT) was used to assess locomotor activity and response to a novel environment. OPT was performed in a custom-made black metal cage (100 cm × 100 cm × 40 cm). The bottom of each cage was divided into 25 equal sectors by strips. Rats were gently placed into the central square and monitored for 5 min. The time spent on grooming, being immobile, as well as that spent rearing and crossing were recorded for each rat. A pre-test was performed before any formal test to allow rats to adapt to the newly faced challenge.

### Forced swimming test

A forced swimming test (FST), designed for evaluation of coping behavior or learning, is widely accepted to assess depression-like behavior through evaluation of the immobility time of the experimental animal in deep water^20,21^. We applied FST to evaluate the effects of propionate on depression. Rats were placed in a transparent glass cylinder containing a 30-cm-depth of water for 6 min. Experiments were videotaped using a ceiling camera and the immobility time for each rat was recorded. Rats were gently dried after the test, and the water was renewed before the next test. A pre-test was performed before any formal test to allow rats to adapt to the newly faced challenge.

### Neurotransmitter quantitation

A panel of 12 neurotransmitters in the hippocampus of each rat was quantified by ultra performance liquid chromatography (UPLC)—electrospray ionization mass spectrometry (ESI–MS/MS) as described previously with minor modifications^22,23^. Briefly, the entire hippocampus of the rats subjected to CUMS modelling and/or propionate administration was homogenized and precipitated with methanol. The supernatant was divided into two aliquots, one aliquot for neurotransmitters quantitation, the other for GC–MS spectrometry. For neurotransmitter quantitation, the supernatant was thoroughly dried with a nitrogen blowing and reconstituted with the initial mobile phase of UPLC. UPLC–ESI–MS/MS was performed with a Thermo Scientific Dionex Ultimate 3000 RSLC system, combined with a Thermo Q Executive Orbitrap mass spectrometer. The analyte was separated using a Thermo Hypersil GOLD (2.1 × 100 mm, 1.7 μm) column. The mobile phase consisting of phase A (water: formic acid (99.9: 0.1, v/v)) and B (acetonitrile: formic acid (99.9: 0.1, v/v)) was applied with a gradient elution at a flow rate of 0.3 ml/min: linear increase from 0% B to 20% B in 3 min; hold at 60% B for 3 min; linear increase from 60% B-80% B in 4 min; linear increase from 80% B to 95% B in 3 min; hold at 95% B for 4 min. ESI–MS/MS conditions were set as follows: gas temperature 350 °C, gas flow 46/min, capillary voltage 3000 V, nebulizer pressure 35 ps. MS acquisitions were performed in the PRM mode.

The calibration curves for each neurotransmitter were obtained by linear regression analysis with 1/x^2^ weighting factor, which contained 10 points covering a linear range of 0.02–20 ng. Data acquisition and analysis were performed with Thermo Xcalibur 2.2 software.

### GC–MS spectrometry

The GC–MS spectrometry of hippocampus samples was conducted as previously described^24^. The aliquoted supernatant of homogenized hippocampus was thoroughly dried by a nitrogen blowing equipment and resuspended in a 30 μl pyridine-methoxy amino acid salt solution (15 mg/ml). The suspension was incubated at 70 °C for 1 h, and then 50 μl N, O-bis (trimethylsilyl) tri-fluoroacetamide (including 1% trimethylchlorosilane) was added into the solution for another incubation at 40 °C for 1.5 h. One microliter of each analyte was injected in a (10:1) split mode into a trace gas chromatograph coupled with a Polyris Q Ion Trap mass spectrometer (Thermo Fisher Scientific, MA, USA). Separation of the ECF derivatives was conducted with a DB-5MS capillary column (30 m × 250 μm i.d., 0.25 μm film thickness, Agilent J & W Scientific, CA, USA). Helium was used as the carrier gas at a constant flow rate of 1.0 ml/min. The oven temperature was first held at 80 °C for 3 min, ramped to 140 °C at a speed of 7 °C/min, held at 140 °C for 4 min, ramped to 180 °C at a speed of 4 °C/min, held at 180 °C for 6 min, then ramped to 280 °C at a speed of 5 °C/min, and finally, held at 280 °C for 2 min.

The mass data was collected in full scan mode from m/z 50 to 650. Compounds were identified by comparison of mass spectra with those in the National Institute of Standards and Technology (NIST) library (version 2.0). The Human Metabolome Database (HMDB) (https://www.hmdb.ca) was employed for further reference. The identified metabolites were validated with commercially available standards. The GC–MS generated raw result files were converted to Net-CDF format and then processed using XCMS with the default settings.

### Metabolome analysis

The metabolomic analysis was performed as previously described^25^. Briefly, the metabolomic dataset was normalized to constant sum and scaled with Pareto scaling using SIMCA-P 13.0 (Umetrics AB, Umea, Sweden). Principal component analysis (PCA) was applied to explore the natural separation among the study groups. Orthogonal Projection to latent structure-discriminate analysis (OPLS-DA) was used to investigate the difference between groups by incorporating the known information of classification. Metabolite with a variable importance for the projection (VIP) value greater than 1 in the established OPLS-DA model, and *P* value < 0.05 in an independent-samples t-test was taken as differential metabolite contributing to the separation of the study groups. Metabolic pathway enrichment of the differential metabolites was performed by the MetaboAnalyst web portal (http:// www.metaboanalyst.ca).

### Statistical analysis

Data of metabolomic profiling, behavior indexing, and neurotransmitters quantitation were expressed as mean ± S.E.M. Nonparametric t-tests were performed in SPSS 22.0 (Chicago, USA) for between-group statistical analyses. A false discovery rate (FDR) was applied as a post-hoc test method for multiple comparisons in metabolomic profiling. *P* < 0.05 was considered statistically significant.

### Ethical approval

All procedures involving experimental animals were in accordance with the Ethical Standards of the institutional and national research committee, and with the 1964 Helsinki Declaration and its later amendments, and were approved by the Ethics Committee of Shanxi University.

## Results

### The anti-/pro- depression effects of propionate were dose dependent

**Figure 2:**
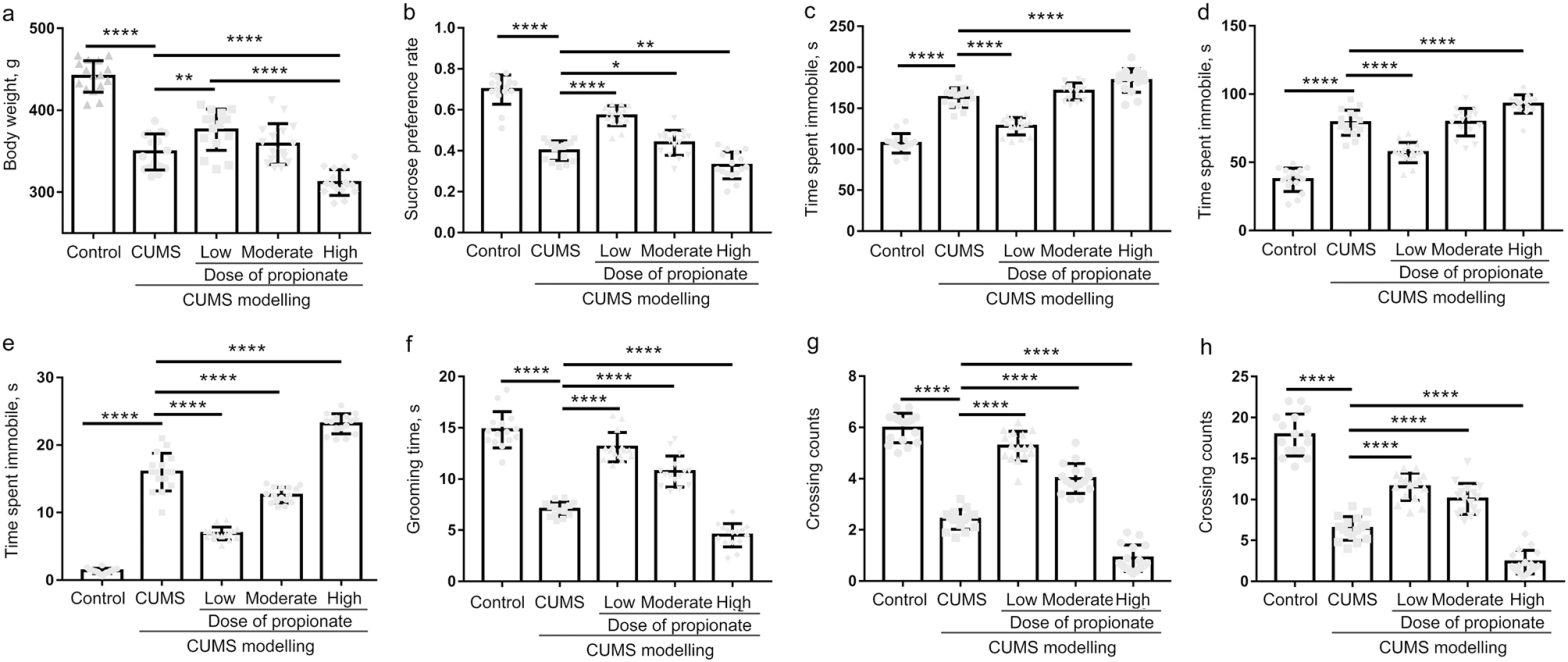
Behavior tests of the CUMS rats intravenously administrated with dose gradients of propionate. Body weight (**a**), sucrose preference rate (**b**), time spent immobile in tail suspension test (**c**), time spent immobile in forced swimming test (**d**), and indices of open field test (**e**–**h**) were recorded or tested with the protocol described in the Material and Methods section at the time point shown in Fig. 1. The data are shown as mean ± S.E.M, with shapes indicating the corresponding value from each rat. A nonparametric t-test was applied for between-group statistical significance. *P < 0.05, **P < 0.01, and ***P < 0.001, ****P < 0.0001.

CUMS is a widely accepted method to model depression-like behaviors in experimental animals, which mimic the social stressors suffered by human beings^19,26^. To evaluate the effects of propionate on depression, we first performed CUMS modelling on rats for four weeks, and then intravenously administrated propionate once per day for one week (Fig. 1). The success of CUMS modelling and the effects of propionate dose gradients on CUMS were evaluated by body weight changes, depression-like behaviors, and by hedonic and reward-seeking behavior. Compared to the healthy controls, the CUMS rats exhibited significantly reduced body weight (Fig. 2a) and reward-seeking behavior (represented by sucrose preference rate, Fig. 2b), and significantly increased depressive-like symptoms (Fig. 2c–h), suggesting the success of the CUMS modelling.

The propionate dose gradients exhibited opposing effects on the CUMS rats. Low-dose propionate (falls into the permitted scope of 3.6–40.8 mg/kg body weight/day^27^) exerted an antidepressant effect, which was demonstrated by recovery of body weight (Fig. 2a) and reward-seeking behavior (Fig. 2b), and significant reduced depressive-like behaviors (Fig. 2c–h). While high-dose propionate exerted a prodepressant effect, which was supported by reduced body weight (Fig. 2a) and reward-seeking behavior (Fig. 2b). High-dose propionate also aggravated depressive-like behaviors (Fig. 2c–h). These results suggest that the double-edged effects of propionate on depression are dose dependent.

### Propionate induced restoration and depletion of neurotransmitters in a dose dependent manner

**Figure 3:**
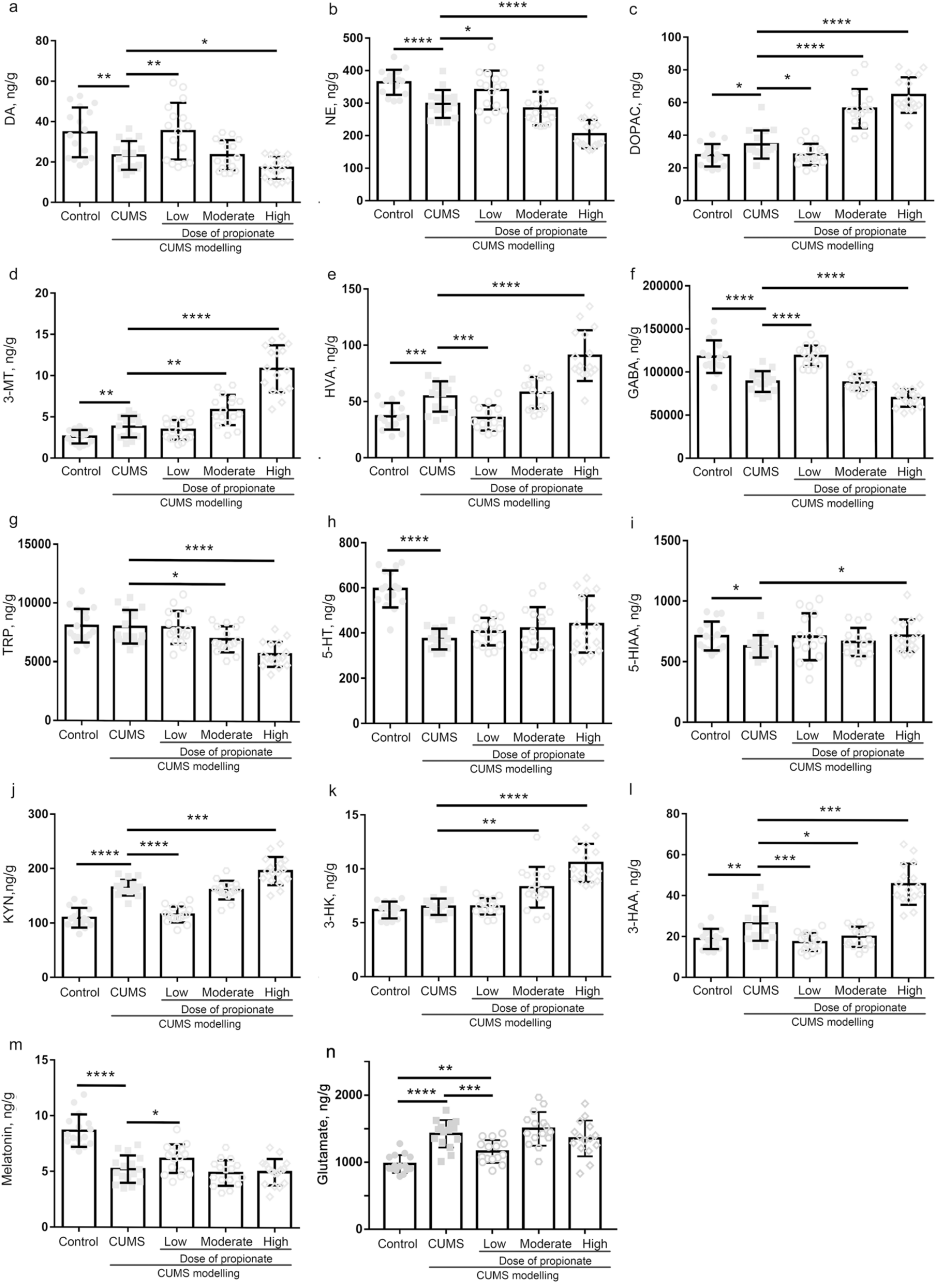
Quantitation of neurotransmitters in the hippocampus of CUMS rats intravenously administrated with dose gradients. Samples of hippocampus were collected after sacrifice of the rats (at the time point shown in Fig. 1) and immediately transferred to liquid nitrogen. Neurotransmitters in the hippocampus of CUMS rats after sacrifice were quantified by HPLC–ESI–MS/MS (Thermo Fisher Scientific, MA, USA). The quantified neurotransmitters included dopamine (**a**), norepinephrine (**b**), dihydroxy-phenyl acetic acid (**c**), 3-methoxytyramine (**d**), homovanillic acid (**e**), gama-aminobyteric acid (**f**), tryptophan (**g**), serotonin (**h**), 5-hydroxyindole acetic acid (**i**), kynurenine (**j**), 3-hydroxykynurenine (**k**), 3-hydroxyanthranilic acid (**l**), and melatonin (**m**). A nonparametric t-test was applied to determine the statistical significance of differences between groups. *P < 0.05, **P < 0.01, and ***P < 0.001, ****P < 0.0001.

The dose dependent contradictory effects of propionate on depression indicated that it might exert opposite modulations on the pathologies of depression. Because neurotransmitters depletion in the central nervous system is a key feature of depression, we next investigate the effects of a propionate dose gradients on neurotransmitters of the hippocampus (Fig. 3). Among the 14 neurotransmitters investigated in this study, NE, DA, 5-HT, 5-HIAA, GABA and melatonin were significantly reduced by CUMS modelling while glutamate, HVA, DOPAC, and 3-MT were prominently increased by CUMS modelling. Among the CUMS reduced neurotransmitters, NE, DA, and GABA were restored by low-dose propionate, while 5-HT, 5-HIAA and melatonin were not significantly affected. All of the CUMS increased neurotransmitters were markedly down-regulated by low-dose propionate. These results support the quick onset antidepressant effects of propionate through selective restoration of NE, DA and GABA, rather than 5-HT. Nevertheless, high dose propionate exhibited opposing effects on these neurotransmitters. High-dose propionate greatly reduced the abundances of NE, DA, 5-HT and TRP, and increased the abundances of GABA, KYN, HVA, 3-HAA, 3-HK, DOPAC, and 3-MT (Fig. 3). These results suggest that restoration and depletion of neurotransmitters by propionate are dose-dependent.

### Hippocampus metabolome profiling reveals altered neurotransmitter metabolism following propionate administration

**Figure 4:**
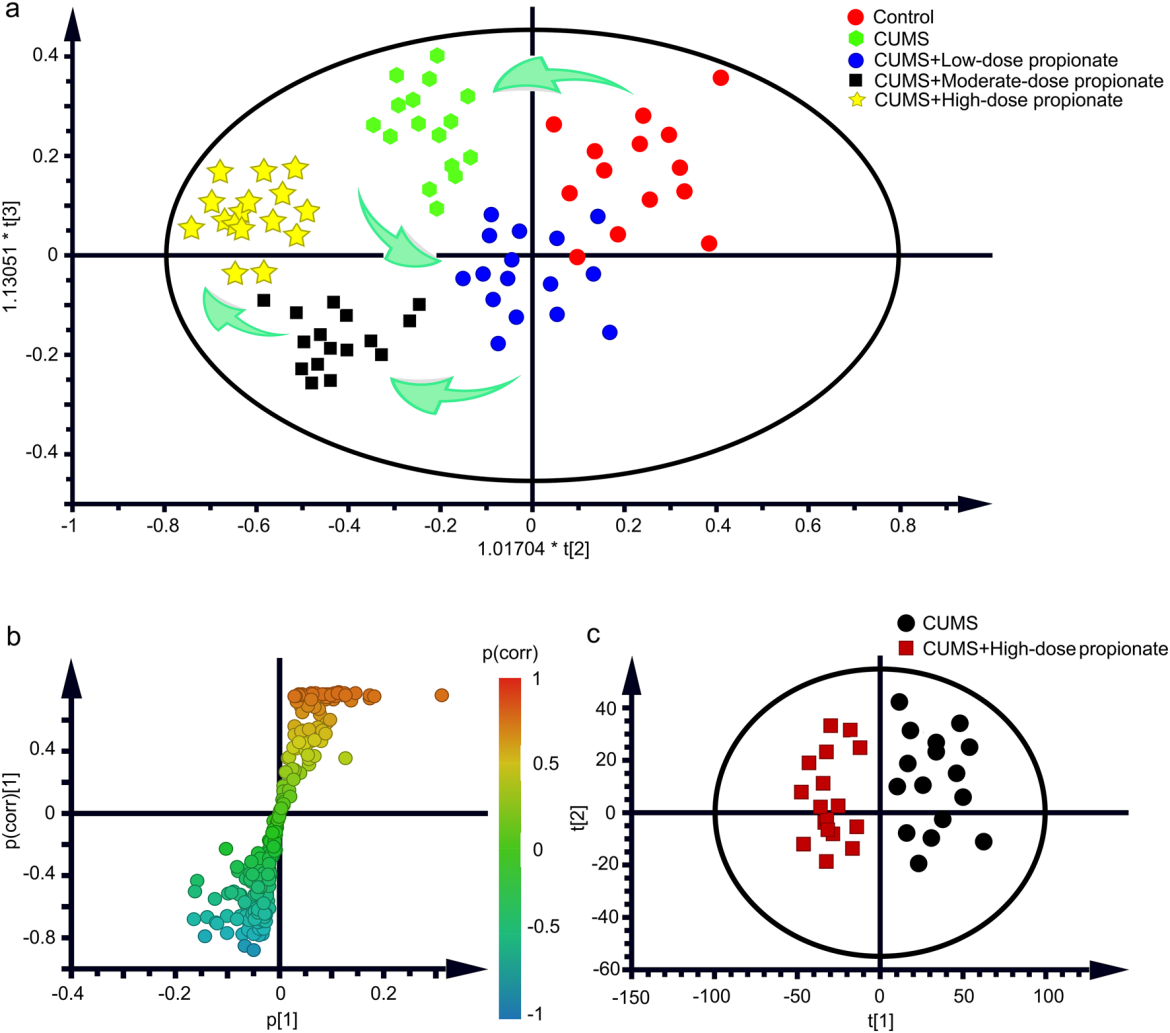
Metabolomic profiling of the CUMS rats receiving propionate administrations. (**a**) Principle component analysis (PCA) of the healthy control rats, the CUMS rats, and the CUMS rats receiving low/moderate/high-dose propionate. Red dots represent the healthy control group, green dots represent the CUMS group. Blue dots represent the group of CUMS rats receiving low-dose propionate. Black blocks represent the group of CUMS rats receiving moderate-dose propionate. Yellow stars represented the group of CUMS rats receiving high-dose propionate. Green arrows represented the trajectories of principle components. (**b**) S-plot analysis between the CUMS group and the CUMS + High-dose propionate group. (**c**) Score plot of Orthogonal Projection to Latent Structures Discriminant Analysis (OPLS-DA) between the CUMS group and the CUMS + High-dose propionate group.

Because several neurotransmitters altered by high-dose propionate were on the same pathway of neurotransmitter metabolism, we speculated that high-dose propionate might induce metabolic disorders in the central nervous system. To confirm the speculated metabolic changes induced by propionate administration, we next performed metabolome profiling on hippocampus of the CUMS rats. The metabolomics experiments were well reproducible (Supplementary Fig. S1a). A good predictive power of the PLS-DA model was assessed by validation plot (Supplementary Fig. S1b). From the principle componenta analysis (PCA) of the metabolome profiles, CUMS modelling induced metabolic disorder in the hippocampus (clear separation from the healthy control group), which was partially rebalanced by low-dose propionate, but aggravated by moderate or high-dose propionate (Fig. 4a). These results suggest that the anti- /prodepressant effects of propionate dose gradients are correlated with the metabolome of the hippocampus.

To explore the key metabolites targeted by propionate further, we next analyzed the differential metabolites between the CUMS group and the group of CUMS rats receiving high-dose propionate from the results of metabolomic profiling. Within the 696 GC–MS spectral features generated from the hippocampus metabolome, 42 metabolites were putatively identified. A total of 10 differential metabolites (Table 1) were further selected by S-Plot analysis (Fig. 4b) and by orthogonal partial least squares discrimination analysis (Fig. 4c), with the thresholds of VIP > 1 and *p* value in an independent sample *t-test* < 0.05. Among the 10 differential metabolites, 3 were neurotransmitters (TRP, GABA, and 3-HAA), 4 were substrates or intermediates of neurotransmitter metabolism. These results demonstrate that high-dose propionate disturbed neurotransmitter metabolism in the hippocampus of the CUMS rats.

**Table 1:** Differential metabolites between the CUMS rats and CUMS rats received high-dose propionate.

Metabolites	Retention time (min)	VIP^a^	*P*-value	Adjusted *P-*value
Propionic acid	9.79	5.62	8.59E−05	1.36E−05
Serine	12.35	6.03	7.75E−06	2.02E−05
Kynurenic acid	13.28	3.14	6.71E−04	7.66E−03
Trans-cinnamic acid	14.25	5.77	4.39E−04	2.15E−03
Quinoline acid	15.60	4.09	4.22E−04	1.96E−03
Aspartic acid	16.28	3.31	6.75E−04	7.80E−03
Tyrosine	26.87	2.08	3.25E−04	6.33E−03
Tryptophan	30.64	4.91	7.66E−06	1.02E−06
GABA	34.29	5.43	2.62E−06	9.78E−05
Phenylalanine	35.96	9.17	3.32E−05	7.63E−04

*VIP* variable importance for projection.

## Discussion

An optimal dose of propionate could produce energy in ruminants and affect various human physiological actions through acting as a precursor for glucose production^28^. However, high-dose propionate may induce autism, suggesting definite adverse effects of propionate to the central nervous system^17,29,30^. As propionate is widely used as a food additive with a permitted scope of 3.6–40.8 mg/kg body weight/day^27^, the cumulative toxicity of propionate to depression is nonnegligible and has not yet been determined. The results of the present study suggest that low-dose propionate (2 mg/kg body weight per day) exerts antidepressant-like effects, but high-dose propionate (200 mg/kg body weight per day) aggravates depression-like behaviors.

Although people with autism are more likely to develop depressive disorders^31,32^, the conclusions drawn from autism are not necessarily applicable to depression, due to the distinct pathogeneses and susceptible populations with depression and autism^33^. While the adverse effects of high-dose propionate on autism have been closely studied^29,30^, its influence on depression remains largely undetermined. The results of this study suggest that low-dose propionate induces beneficial effects to alleviate depression-like symptoms, but high-dose propionate induces the opposite effect, aggravating depression-like symptoms in CUMS rats. Given the reported antidepressant effects of low-dose propionate^14^, this study is the first report on the prodepressant effects of high-dose propionate.

Disturbances of neurotransmitters are the major adverse effects of propionate in autism^17,34^. High-dose propionate-induced autism is characterized by synchronized depletion of GABA, 5-HT, DA, NE and TRP^35^. Most of these neurotransmitters were depleted by high-dose propionate administration in the animal models of depression in this study, while 5-HT was not significantly affected. These results are consistent with previous reports that 5-HT was not affected by short term administration of propionate and was associated with the slow actions of selective serotonin reuptake inhibitors (SSRIs) antidepressants^36^. Through a comprehensive profiling of neurotransmitters in the hippocampus of depressed rats, KYN, 3-HK, DOPAC, and 3-MT were significantly increased by high-dose propionate in this study. The opposite direction of neurotransmitters induced by high-dose propionate suggests metabolic disturbances in the hippocampus. Hippocampus metabolome profiling in this study demonstrated that metabolism of neurotransmitters was significantly altered by high-dose propionate. In accordance with the prodepressant effects of high-dose propionate, the metabolic profile in the hippocampus was also disturbed (Fig. 4a), characterized by ten metabolites with altered abundances (Table 1). Among the altered metabolites, quinoline acid and kynurenic acid are downstream metabolites of tryptophan. The altered abundances of these three metabolites indicate the disorder of tryptophan metabolism in hippocampus, consistent with findings of previous reports^25,37,38^ Kynurenic acid was reported to be positively correlated with subiculum volume in MDD patients^39^, while quinolinic acid was previously correlated to markers of inflammation in depressed patients^39^. Tyrosine, phenylalanine, and tryptophan are monoamine precursors, simultaneous depletion of which has been used to probe brain monoamine function^40^. Therefore, the altered metabolites observed in the present study, primarily consisting of precursors of neurotransmitters or metabolites of these precursors, suggest that neurotransmitters metabolism in the hippocampus was disturbed by high-dose propionate, which may contribute to the dysbiosis of neurotransmitters.

Given the antidepressant effect of low-dose propionate and the prodepressant effect of high-dose propionate, moderate-dose propionate seems to exert complicated effects in depression. Moderate-dose-propionate acts against CUMS-modelling in open field test (Fig. 2e–h), with no significant effects to body weight, sucrose preference and immobility time in FST and TST (Fig. 2a–d). With respect to the neurotransmitters in the hippocampus, moderate-dose propionate aggravated the CUMS induced dysbiosis of DOPAC, 3-MT and TRP (Fig. 3c,d,g), and restored the dysbiosis of 3-HAA (Fig. 3i). Because low-dose propionate restored the dysbiosis of NE, DA, GABA, DOPAC, HVA, GABA, KYN, 3-HAA and melatonin, it seems that different action pathways underly the effects of low-dose and moderate-dose of propionate. Further studies are recommended to dissect the complicated effects of moderate-dose propionate on depression.

Revealing the roles of propionate in the prefrontal cortex or other brain areas, it is essential to comprehensively understand the underlying mechanism of the dose dependent antidepressant or prodepressant effects of propionate, which is a potential limitation of the present study. As another limitation of this study is the evaluation of the expression and activity of rate-limiting enzymes or vesicular transporters involved in these neurotransmitters synthesis and metabolism. It would be valuable to reveal the underlying mechanism of the dose-dependent effects of propionate in depression. Because sodium propionate is used as a food additive consumed by the general population, its potential adverse effects to homeostasis of neurotransmitter in healthy individuals should be investigated in future studies. Although FST is widely accepted to evaluated the CUMS modelling of depression^20,21^, it is unclear whether FST is suitable to reflect depression^41,42^, as it is not clear whether the animals stop swimming because they are despondent or because they have learned that they will be scooped out of the tank when they stop moving. Thus, FST should be evaluated further for CUMS modelling of depression as should the effects of propionate in future studies. Because some of the metabolites observed in the hippocampus also exist in blood, brain perfusion prior to neurotransmitter profiling could be helpful to determine the contribution of blood to the altered abundances of metabolites observed in the hippocampus.

In summary, this study reports for the first time the adverse effects of high-dose propionate on depression, leveraging an animal model with depressive-like symptoms. The prodepressant effect of high-dose propionate correlated with changes in the abundances of neurotransmitters in the hippocampus, including with the depletion of NE, DA, 5-HT and TRP, and with the increase of GABA, KYN, HVA, 3-HAA, 3-HK, DOPAC, and 3-MT. Metabolomic profiling of the hippocampus supported the metabolic disturbances of neurotransmitters in the hippocampus of CUMS rats receiving high-dose propionate. The results of this study point to a potential cumulative toxicity of propionate as a food additive in mood disorders.

## Supplementary information

Supplementary Figure 1.

Supplementary Information 1.

Supplementary Figure legends.
