# Supplementary figures and images for: Intravenous administration of sodium propionate induces antidepressant or prodepressant effect in a dose dependent manner
Source: Sci Rep. 2020 Nov 16;10:19917. doi: 10.1038/s41598-020-77085-z (PMC7670463; doi:10.1038/s41598-020-77085-z)

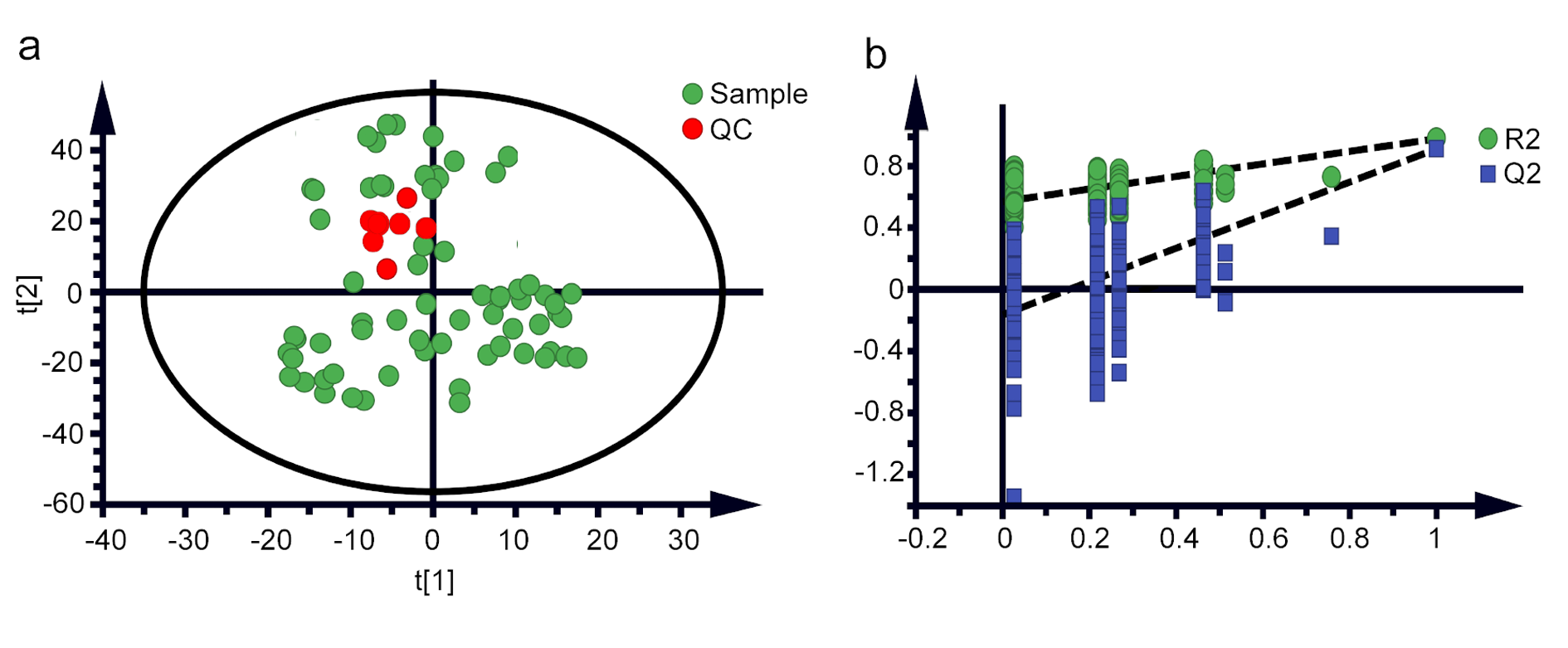

Supplement: Supplementary file 1 — Supplementary Figure 1. [file 41598_2020_77085_MOESM1_ESM.tif]
