# Supplementary material for: Intravenous administration of sodium propionate induces antidepressant or prodepressant effect in a dose dependent manner
Source: Sci Rep. 2020 Nov 16;10:19917. doi: 10.1038/s41598-020-77085-z (PMC7670463; doi:10.1038/s41598-020-77085-z)
Supplement: Supplementary file 2 — Supplementary Information 1. [file 41598_2020_77085_MOESM2_ESM.pdf]

Intravenous administration of sodium propionate induces antidepressant or prodepressant effect in a dose dependent manner

Chunyan Hao[1, #], Zefeng Gao[2, #], XianJun Liu[1], Zhijiang Rong[1], Jingjing Jia[3], Kaiqi Kang[3], Weiwei Guo[3], Jianguo Li[2, *]

1 School of Chemical and Biological Engineering, Taiyuan University of Science & Technology, Taiyuan 030021, China

2. Key Laboratory of Chemical Biology and Molecular Engineering of Ministry of Education, Institutes of Biomedical Sciences, Shanxi University, Taiyuan 030006, China

3. School of Life Science, Shanxi University, Taiyuan 030006, China

# These authors contributed equally to this work.

***Corresponding author**

Dr. Jianguo Li

Key Laboratory of Chemical Biology and Molecular Engineering of Ministry of Education, Shanxi University, No. 92, Wucheng Road, Xiaodian District, Taiyuan 030006, Shanxi, China. Tel/Fax: +86-351-7018958, Email: lijg@sxu.edu.cn.

Author contributions statement

Conception and design: JL, and CH; Execution and data acquisition: CH, ZG, XL, KK and WG; Analysis and interpretation: CH, ZR, JJ and JL; Drafting the manuscript for important intellectual content: CH, ZG, and JL. All authors read and approved the final manuscript.

Running title: The dose-dependent pro-/antidepressant effects of propionate
