# Supplementary material for: Intravenous administration of sodium propionate induces antidepressant or prodepressant effect in a dose dependent manner
Source: Sci Rep. 2020 Nov 16;10:19917. doi: 10.1038/s41598-020-77085-z (PMC7670463; doi:10.1038/s41598-020-77085-z)
Supplement: Supplementary file 3 — Supplementary Figure legends. [file 41598_2020_77085_MOESM3_ESM.docx]

**Supplementary Figure 1. (a) Evaluation of the metabolomic data acquisition quality by PCA of QCs and hippocampus samples.** QCs were prepared through pooling equal aliquots of loading sample for GC-MS spectrometry. The first six QCs were tested before the total analysis to stabilize the analytical system, and the acquired results were removed prior to data processing. PCA was carried out with SIMCA-P 14.0 after total peak area normalization. Each dot represented one QC (red) or one sample (green). **(b) Model validation of the metabolomic analysis.** The model validation analysis was performed by pair-wise comparisons of the hippocampus metabolome between the CUMS group receiving low-dose propionate and the CUMS groups receiving high-dose propionate. A 200 permutation was applied for the validation analysis.
